# Development of a project for interprofessional collaboration between medical and pharmacy students to improve medication safety in polypharmacy (PILLE)

**DOI:** 10.3205/zma001585

**Published:** 2023-02-15

**Authors:** Sabine Gehrke-Beck, Maike Petersen, Wolfram J. Herrmann, Nicole Zimmermann, Eva Daub, Johanna Seeger, Josefine Schulz, Constanze Czimmeck, Noemi Lauterbach, Harm Peters, Charlotte Kloft, Martin Schulz, Ingo Siebenbrodt, Ronja Behrend

**Affiliations:** 1 Charité - Universitätsmedizin Berlin, Charité Campus Mitte, Institut für Allgemeinmedizin, Berlin, Germany; 2 Freie Universität Berlin, Institut für Pharmazie, Berlin, Germany; 3 ABDA - Bundesvereinigung Deutscher Apothekerverbände e. V., Berlin, Germany; 4 Charité - Universitätsmedizin Berlin, Berlin, Germany; 5 Charité - Universitätsmedizin Berlin, Dieter Scheffner Fachzentrum für medizinische Hochschullehre und evidenzbasierte Ausbildungsforschung, Berlin, Germany; 6 Charité - Universitätsmedizin Berlin, Prodekanat für Studium und Lehre, Semesterkoordination Modellstudiengang Medizin, Berlin, Germany

**Keywords:** interprofessional education, polypharmacy, patient safety, distance education, teaching

## Abstract

**Aim:**

Interprofessional collaboration is particularly relevant to patient safety in outpatient care with polypharmacy. The educational project “PILLE” is meant to give medical and pharmacy students an understanding of the roles and competencies needed for cooperation in the provision of healthcare and to enable interprofessional learning.

**Method:**

The curriculum is aimed at pharmacy and medical students and was developed in six steps according to the Kern cycle. It is comprised of an interprofessional seminar, a joint practical training in a simulated pharmacy, and a tandem job shadowing at a primary care practice. The project was implemented in three stages due to the pandemic: The interprofessional online seminar based on the ICAP model and the digital inverted classroom was held in the 2020 winter semester; the interprofessional practical training was added in the 2021 summer semester; and the interprofessional tandem job shadowing at a primary care practice in the 2021 winter semester. Attitudes toward interprofessional learning, among other things, was measured in the evaluation using the SPICE-2D questionnaire (Student Perceptions of Physician-Pharmacist Interprofessional Clinical Education).

**Results:**

In the first three semesters, a total of 105 students (46 pharmacy, 59 medicine) participated in the project, of which 78 participated in the evaluation (74% response rate). The students stated, in particular, that they had learned about the competencies and roles of the other profession and desired additional and more specific preparatory materials for the course sessions. The SPICE-2D questionnaire showed high values for both groups of students already in the pre-survey and these increased further as a result of the project.

**Conclusion:**

Joint case-based learning could be implemented under the conditions imposed by the pandemic. Online teaching is a low-threshold means to enable interprofessional exchange.

## Zusammenfassung

**Zielsetzung:**

In der ambulanten Versorgung von Patient*innen mit Polypharmazie ist interprofessionelle Zusammenarbeit für die Patientensicherheit besonders relevant. Das Lernprojekt PILLE soll Rollenverständnis und Kompetenzen für eine kooperative Versorgung an Pharmazie- und Medizinstudierende vermitteln und interprofessionelles Lernen ermöglichen.

**Methodik:**

Das Curriculum wurde nach dem Kern-Zyklus in sechs Schritten entwickelt und umfasst ein interprofessionelles Seminar, eine gemeinsame Fallbearbeitung in einer Simulationsapotheke und eine Hospitation in einer Hausarztpraxis für Pharmazie- und Medizinstudierende. Pandemiebedingt wurde das Lernprojekt in drei Stufen implementiert: im Wintersemester 2020 das Online-Seminar basierend auf dem ICAP-Modell und dem Konzept des digitalen inverted classroom, im Sommersemester 2021 ergänzend das interprofessionelle Praktikum und im Wintersemester 2021 die Hospitation in einer Hausarztpraxis. In der Evaluation wurde u.a. die Einstellung zu interprofessionellem Lernen mit dem SPICE-2D Fragebogen (Student Perceptions of Physician-Pharmacist Interprofessional Clinical Education) erhoben.

**Ergebnisse:**

In den ersten drei Semestern nahmen insgesamt 105 Studierende (46 Pharmazie, 59 Medizin) am Lernprojekt teil, davon beteiligten sich 78 an der Evaluation (74% Rücklauf). Die Studierenden geben an, besonders zu Kompetenzen und Rolle der anderen Berufsgruppe gelernt zu haben und wünschen sich weitere gezielte Vorbereitungsmaterialien für den Unterricht. Der SPICE-2D Fragebogen zeigt bereits vorab hohe Werte bei beiden Studierendengruppen, die sich durch das Lernprojekt weiter erhöhten.

**Schlussfolgerung:**

Gemeinsames fallbasiertes Lernen war unter Pandemiebedingungen umsetzbar. Online-Lehre bietet eine niedrigschwellige Möglichkeit, interprofessionellen Austausch zu ermöglichen.

## 1. Introduction

As a result of demographic change, polypharmacy is receiving increased attention as a problem regarding patient safety: 42% of all people over 65 in Germany take more than five medications per day [1]. Often these are prescribed by different physicians and bought at different pharmacies. Polypharmacy is associated with adverse drug effects, over- and under-prescribing, and increased hospital admissions [2], [3], [4]. Coordination between physicians and pharmacists could contribute to an improvement in patient care. However, collaboration between these professional groups is not sufficiently addressed or trained during medical or pharmacy study.

The teaching and learning project “PILLE” (*Collaboration between Medical and Pharmacy Students to improve Medication Safety in Polypharmacy: An Interprofessional Teaching and Learning Project)* was developed to teach competencies for better interprofessional communication and collaboration, in that medical and pharmacy students learn cooperatively during their studies about multimorbidity and medication safety.

### 1.1. Problem identification and setting the objective

Patients and physicians have reported problems regarding the care of multimorbid patients due to difficulties in interprofessional communication and a lack of time [5], [6], [7]. Improved interprofessional collaboration has a positive influence not only on the frequency of errors and patient satisfaction, but also on job satisfaction in the healthcare professions [8], [9].

In order to train for interprofessional collaboration already during university study, interprofessional education will be needed for all healthcare professions, and such an approach is provided for in the drafts of the new medical licensing regulations [10], [11], [12], [13], [https://www.nklm.de]. Communication techniques for interacting with physicians and members of other healthcare professions are also explicitly mentioned as educational content in the licensing regulations for pharmacists [https://www.gesetze-im-internet.de/aappo/anlage_8.html]. However, due to the monoprofessional organization of education and training in the healthcare professions, interprofessional education is still inadequately implemented.

In primary care, teamwork is seen as relevant to patient-centered care and, at the same time, felt to be challenging [14]. Professionals who provide medical care for the same patient often work in separate outpatient settings, and high workloads make establishing contact between them more difficult. While at the same time, the vast majority of patient care and medication is provided in the outpatient primary care setting [15]. By contrast, current interprofessional learning courses have been implemented mainly in the inpatient setting at university hospitals because that is where most of the clinical training is offered for medical students. PILLE is a project meant to create an interprofessional learning course in the outpatient sector.

## 2. Project description

Setting: The project was undertaken in cooperation between the new revised medical curriculum at the Charité – Universitätsmedizin Berlin (Charité) and the pharmacy degree program at the Freie Universität Berlin (FU Berlin). The project team included teachers and students from both degree programs, as well as university staff in education research and course scheduling, and was cooperatively organized [16]. The project was designed in accordance with Kern's six steps for curriculum development ([17], referred to in the following as steps 2 through 6). Step 1, “problem identification”, is described above in section 1.1.

### 2.1. Step 2: “Targeted needs assessment”

Pharmacy students in the 6^th^ to 8^th^ semester of study at the FU Berlin are participating voluntary in the project and randomly selected seminar groups of 10^th^-semester medical students at the Charité participated as part of a compulsory seminar, in the module on general medicine, emergency medicine, paperwork and interfaces. Both groups of students have only limited prior experience with interprofessional courses:

No interprofessional courses are offered in FU Berlin’s pharmacy degree program. In the 6^th^-8^th^ semester a required elective on “patient-centered pharmacy” is offered, in which communication withphysicians is covered.

A longitudinal curriculum for interprofessional education is being developed for Charité’s medical degree program with shared learning opportunities for study programs in different healthcare professions (e.g., B.A. in nursing, B.A. in applied midwifery). The first teaching formats have already been implemented, e.g., a scheduled time for interprofessional teamwork in the communication curriculum for the 5th semester of the new revised medical curriculum [18]. The topic is taught to all students of the semester, but only for approximately 10% of the students, in interprofessional groups with nursing students. Joint learning with pharmacy students has not previously taken place. A survey of Charité medical students showed that they rate the relevance of interprofessional education very highly [19].

#### 2.2. Step 3: “Goals and objectives”

PILLE is intended to build on limited prior experience by discussing the roles of general practitioners and pharmacists in providing patient care and teach the practical aspects of working as a team based on patient cases in a simulated setting (medication management center at the FU) and in the routine clinical setting (block practicum in general medicine at primary care practices).

The project group formulated learning objectives that build upon the prior knowledge of both student groups. The defined learning objectives are listed in table 1 (Tab. 1).

#### 2.3. Step 4: “Educational strategies”

##### 2.3.1. Strategic concept

PILLE is offered in the final semesters of study when the students in both subject areas already have sufficient competencies to jointly plan and discuss patient-centered therapy.

**Figure 1:**
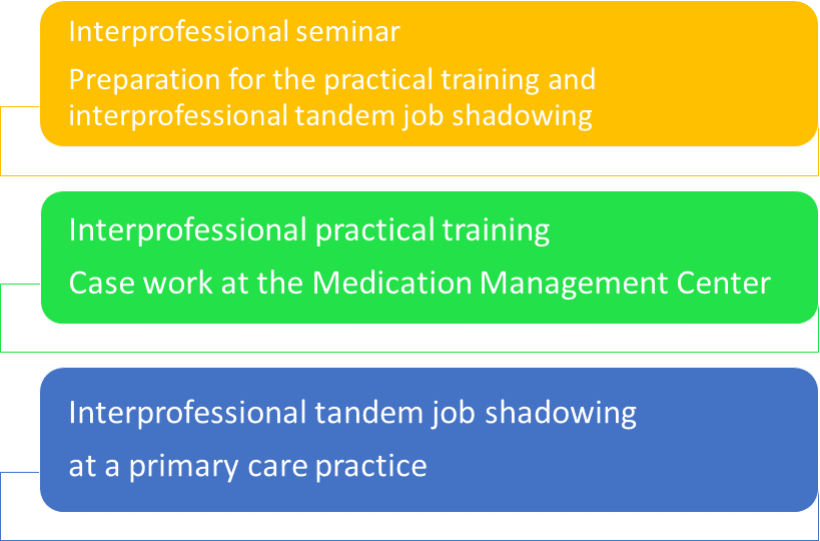
General concept of the project

With constructive alignment in mind, courses were designed based on learning objectives with which attitudes could be reflected and skills taught [20]. Teaching formats were designed which initially covered theoretical and then practical interprofessional case discussions and simulations. Interprofessional teamwork can thus be experienced, practiced and reflected upon using concrete examples. The project is comprised of three parts (see figure 1 (Fig. 1)):

1. The students become acquainted with each other in an *interprofessional seminar* and discuss a case with drug-related problems in small interprofessional groups.

The two practicums following provide opportunities to engage in and practice teamwork in the relevant professional context:

1. A medication review is undertaken in an *interprofessional practical training* in a simulated pharmacy (medication management center at the FU Berlin) and a patient consultation is conducted in small interprofessional groups.
2. Students jointly hold a medication review and a patient consultation as part of the *interprofessional tandem job shadowing* at a primary care practice within the scope of the block practicum in general medicine. As a part of this, the pharmacy students accompany the medical students for half a day at the primary care practice.

##### 2.3.2. Interprofessional seminar

Due to the pandemic, the seminar was scheduled as a live online course. Two theoretical concepts were taken into account in order to organize the online course effectively:

The *ICAP (interactive, constructive, active, passive) framework* [21] assumes that learning can most effectively take place if active, constructive and interactive learning activities are also included instead of just purely passive learning activity. Among these types, interactive learning activities are ranked as the most effective. Therefore, our aim for the seminar was to include a case analysis by a small interprofessional group as the central learning element and to plan sufficient time for this activity.

For this reason, the *inverted classroom model for online teaching* [22] was used. This model uses actual classroom time for in-depth discussions and answering questions, while any knowledge content is imparted in advance by means of individual preparation. Course content involving only the acquisition of knowledge took the form of asynchronous e-learning units available online. For this, two teaching videos were created on multimorbidity and medication safety, and students were asked via email to watch them in advance as preparation. In terms of the content, strategies for medication review and de-prescribing were, shared decision-making in multimorbidity, and the “swiss cheese model” of medication safety.

The seminar was team taught by lecturers from general medicine and clinical pharmacy so as to include the perspectives of both professional groups.

##### 2.3.3. Interprofessional practicums

The practical training at the medication management center (MMC) was held in person. Medical and pharmacy students were paired together to conduct a medication review with one or more case examples using the scientific literature, internet, and pharmacy software. Afterwards, a patient consultation was held as a role play and discussed in a feedback round with two teachers from both professional groups.

As part of the tandem shadowing at the primary care practice, the students jointly queried a patient, analyzed the medication plan, and conferred about potential drug-related issues with the teaching physician.

##### 2.4. Step 5: “Implementation”

The project was implemented in stages due to the pandemic: the interprofessional seminar was held as a live webinar in the 2020/21 winter semester. It was possible to add the interprofessional practical training in the 2021 summer semester as an in-person course at the MMC, in compliance with the necessary hygiene measures. Starting with the 2021/22 winter semester, the third element of tandem job shadowing at the primary care practices was implemented.

##### 2.5. Step 6: “Evaluation”

The evaluation was designed according to the Kirkpatrick model, in which participant satisfaction and the effects of the teaching on learning and behavior are measured [23]. Regarding satisfaction with the course, direct questions were asked about how the students rated the seminar’s structure with its emphasis on discussion and sharing and whether they had prepared in advance for the seminar. Suggestions for improvement were elicited by asking open-ended questions.

Learning success was measured by means of subjective evaluation by the participants using a 5-point Likert scale and open-ended questions. Attitudes toward interprofessional learning were captured by the validated SPICE-2D questionnaire (Student Perceptions of Physician-Pharmacist Interprofessional Clinical Education, version 2, German, see table 2 (Tab. 2)) before and after the project [24], [25]. The questionnaire measures attitudes toward interprofessional education, whereby higher values (parameters 1-5) stand for a more positive attitude (see attachment 1 ). The evaluation was carried out online; the link to participate was sent to the participants before and after the project.

## 3. Results

### 3.1. Implementation and participants

In the first three semesters of implementation, a total of 105 students (46 pharmacy, 59 medicine) participated in the project see table 3 (Tab. 3)). During the 2020/21 winter semester one seminar group participated in the model curriculum; due to the large amount of interest, the project was continued with two seminar groups starting in the 2021 summer semester.

In the 2020/21 winter semester only a few medical students participated because the seminar time slot conflicted with the mandatory corona tests for students. Participation levels came back up in the following semesters.

#### 3.2. Evaluation results

Overall, during the first three semesters 78 of 105 students participated in the evaluation (74% response rate).

##### 3.2.1. Seminar

The first time the seminar was offered, 14 of the 19 participants took part in the evaluation (74% response rate). All 14 students said that they would recommend the seminar to others. All but one of the 14 students stated that they had prepared themselves in advance for the seminar. Three students reported a desire for more input, 11 desired more time for sharing. The students commented in the free texts that they had gained insights into the other professional perspective, competencies and approaches (n=8). Comments and suggestions for improvements included having more time to share information and more interprofessional seminars in the study program, more specific opportunities to prepare, and more information about routine professional cooperation and communication between medical practices and pharmacies.

**Figure 2:**
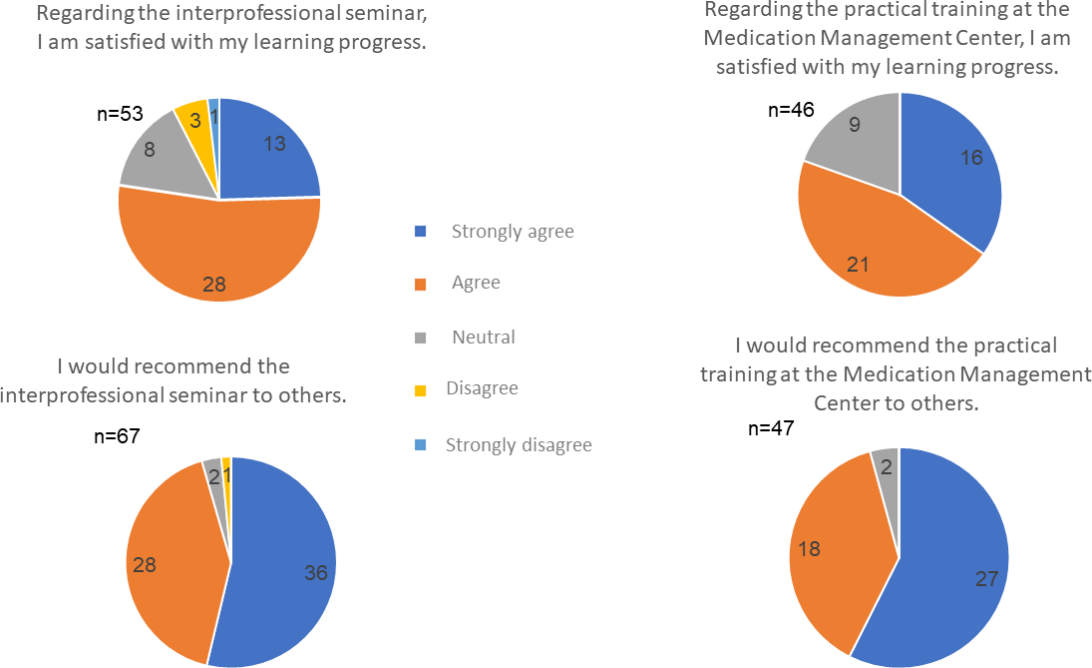
Feedback from the post-survey for 3 cohorts of PILLE: 2020/21 winter semester, 2021 summer semester, 2021/22 winter semester. a) satisfaction with the learning progress. b) recommendation to others

Satisfaction was also high in the two subsequent semesters; 50 of 53 evaluating students said they would recommend the seminar to others (2 partly yes/partly no, 1 no), and 41 of 53 were satisfied with their learning progress (8 partly yes/partly no, 4 no) (see figure 2 (Fig. 2)).

The following positive aspects were identified in the free-text comments: friendly atmosphere, familiarization with the perspectives of the other professional group, working through cases in small groups, changing from a large group to small groups, sharing in general, and how to handle uncertainty as a topic.

##### 3.2.2. Interprofessional practicums

Of the 64 participants in the MMC practical training, 46 took part in the evaluation (71% response rate). A total of 45 out of 46 stated that they would recommend the MMC practical training to others, and 37 were satisfied with their learning progress during the practical training (9 partly yes/partly no) (see figure 2 (Fig. 2)). Students rated the joint case work, the small groups, the follow-up discussions, and the role playing positively in the free-text comments. Suggestions for improvement included the observation that the role-playing exercises could be prepared for at home in advance and, in addition to a simulated patient consultation, a simulated telephone call between attending primary care physicians would be helpful.

A total of 26 participants evaluated the job shadowing at the medical practices: Of these, 13 students reported that the shadowing could not take place, usually for organizational reasons. Thirteen students evaluated an actual job shadowing experience. Eight were satisfied with their learning progress (4 partly yes/partly no, 1 no), and nine of 11 would recommend the tandem shadowing to others (2 partly yes/partly no). In the free-text comments, positive reports were made that it had been possible to advise eight patients in real clinical contexts, and the follow-up discussions with experienced physicians were viewed positively. Using unfamiliar software in the medical practices was identified as difficult.

#### 3.3. Attitude toward collaboration (SPICE-2D)

**Figure 3:**
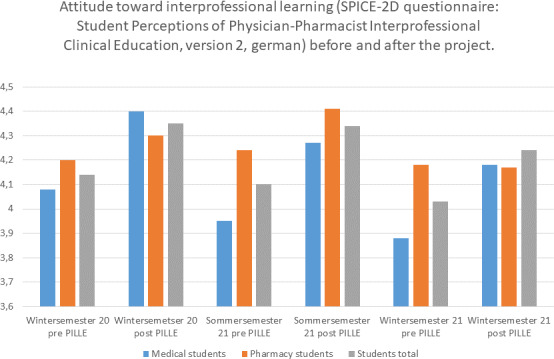
Attitudes toward interprofessional learning (SPICE-2D questionnaire: Student Perceptions of Physician-Pharmacist Interprofessional Clinical Education) before and after the project

Already before taking the interprofessional course, students' attitudes were high on the 5-point scale at 4.14 (semester 1), 4.10 (semester 2) and 4.03 (semester 3) and climbed further to 4.35, 4.34 and 4.24 as a result of the project (see figure 3 (Fig. 3)). Here, it is seen that pharmacy students have a somewhat more positive attitude in the beginning and that medical students experience a larger change in their attitude.

## 4. Discussion

### 4.1. Summary

It was possible to feasibly implement the interprofessional education project in an adapted format under pandemic conditions. After just one online course in the 2020/21 winter semester, students reported a gain in knowledge about the competencies and perspectives of the other professional group and voiced support for intensive communication and cooperation between the professional groups. In-depth sharing was fostered and promoted in the online format through an inverted classroom strategy. Most students indicated that they had prepared themselves for the seminar, and many students desired more preparatory and supplemental materials.

#### 4.2. Consequences of the evaluation

In the evaluation the participants expressed a desire especially for more specific opportunities to prepare for the joint learning sessions. This was surprising because at first it was unclear if the students would even use the preparatory materials in the context of the inverted classroom since neither required preparation nor blended learning concepts are common in either degree program. The preparatory materials were then supplemented, and case descriptions and assignments were made accessible for the purpose of more focused preparation.

Based on the feedback for the practical training at the MMC, the joint practicum was structured more effectively and clear role instructions and materials (medication plan, prescription samples, etc.) were created for the role playing.

#### 4.3. Strengths and weaknesses

This project implements an interprofessional teaching strategy regarding a topic relevant to healthcare and patient safety and thus offers an opportunity to prepare for interprofessional learning in anticipation of the new medical licensing regulations. Since interprofessional learning for pharmacy and medical students is still only rarely implemented, it was possible to gather experience that can be of help to other educational institutions.

A cooperatively organized planning group is recommended for the implementation of interprofessional education. For this project, such an organizational structure was not only able to successfully coordinate the course planning, but also respond adequately to the challenges posed by the pandemic and coordinate the rescheduling which was then implemented by both faculties. The implementation of the seminar in an online format arose as an emergency solution, which will also be used in future to enable low-threshold participation and attendance without requiring travel to and from external campus locations.

One weakness of the project is the lack of funding since the additional teaching and planning efforts going into interprofessional education are currently undocumented. The planning and implementation were done by project staff in addition to their regular duties and is therefore dependent on an above-average commitment to teaching and education. Adequate funding of the human resources as required by the very nature of interprofessional education is urgently needed.

Another disadvantage is that the project did not entail an assessment specifically to test the learning objectives. The rules and regulations governing the new revised medical curriculum do not provide for an assessment in the 10^th^ semester and because of this it was not organizationally viable to objectify learning success by means of a test. In terms of constructive alignment, though, as an example, an OSCE station with an interprofessional case conference focused on a medication review would have been meaningful.

The high values measured by the SPICE-2D instrument at the start limit the meaningfulness of the change resulting from the project; however, these constant improvements could have possibly arisen as an intervention effect since one limitation regarding the results to date is that there has been no comparison group.

#### 4.4. Continuation and further development of the project

PILLE will continue and is already offered as part of the required curricular coursework in the medical degree program. This requirement is planned for the pharmacy students starting in the 2022 summer semester. An expansion of the project to include all students at a specific semester level in the model medical curriculum is unfortunately infeasible due to the lower numbers of students enrolled in the pharmacy program. As an alternative, it may be possible to integrate other professional groups into this study phase with a focus on other major topics.

## 5. Conclusions

The experience gathered in this project shows that it is possible, with specific planning, to gain insights into other professional points of view and reflect on roles even in an online classroom. The advantage here is that scheduling times and classrooms for more than one university and commuting to another campus are not necessary. Practical exercises to learn team-related skills are indispensable and can be prepared for in specific ways. By applying a flipped classroom model to interprofessional education, it is possible to address the different kinds of prior knowledge that the participants bring with them and to optimally use the joint sessions by ensuring advance individual preparation.

This project can support the implementation of the new medical licensing regulations as a model for the development of teaching at outpatient medical practices and the implementation of interprofessional education in a context relevant to patient safety.

## Acknowledgements

We wish to thank the Lesmüller Stiftung for the funding to carry out the evaluation and Yvonne Pudritz et al. for the use of the German translation of the SPICE-2D questionnaire.

## Competing interests

The authors declare that they have no competing interests. 

## Supplementary Material

Evaluation survey

## Figures and Tables

**Table 1:**
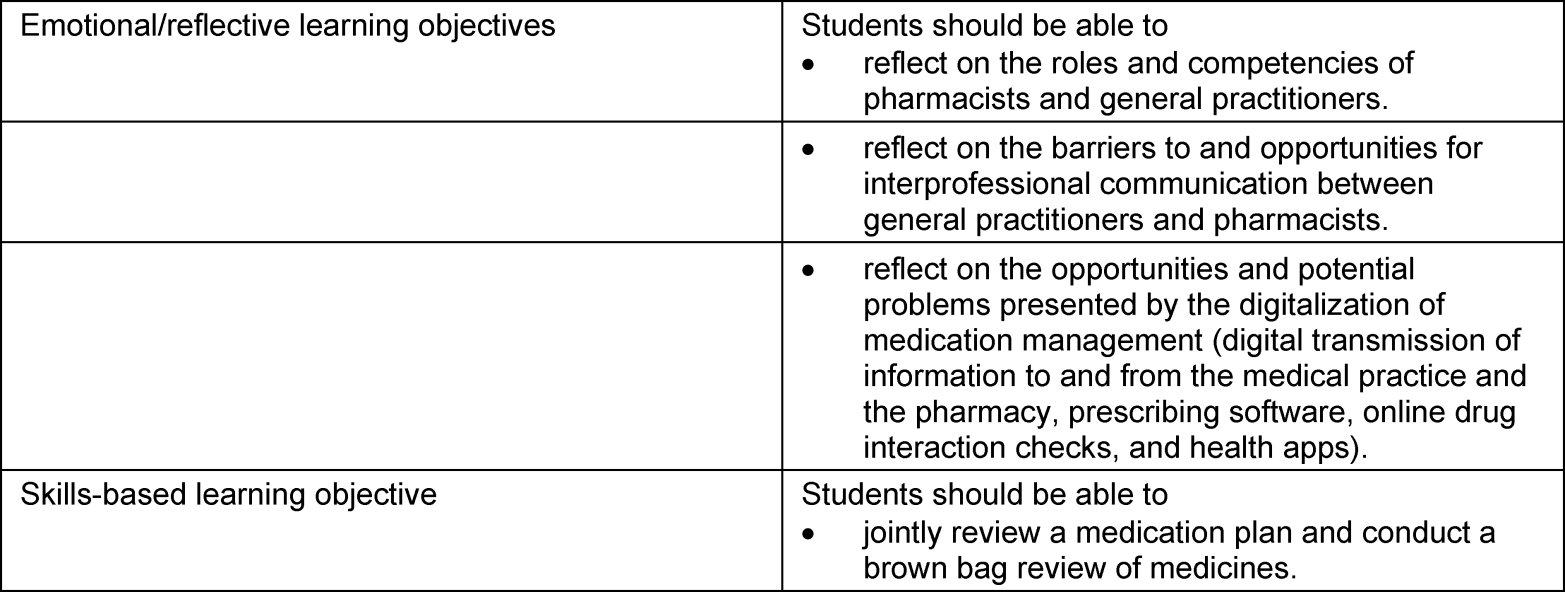
PILLE learning objectives

**Table 2:**
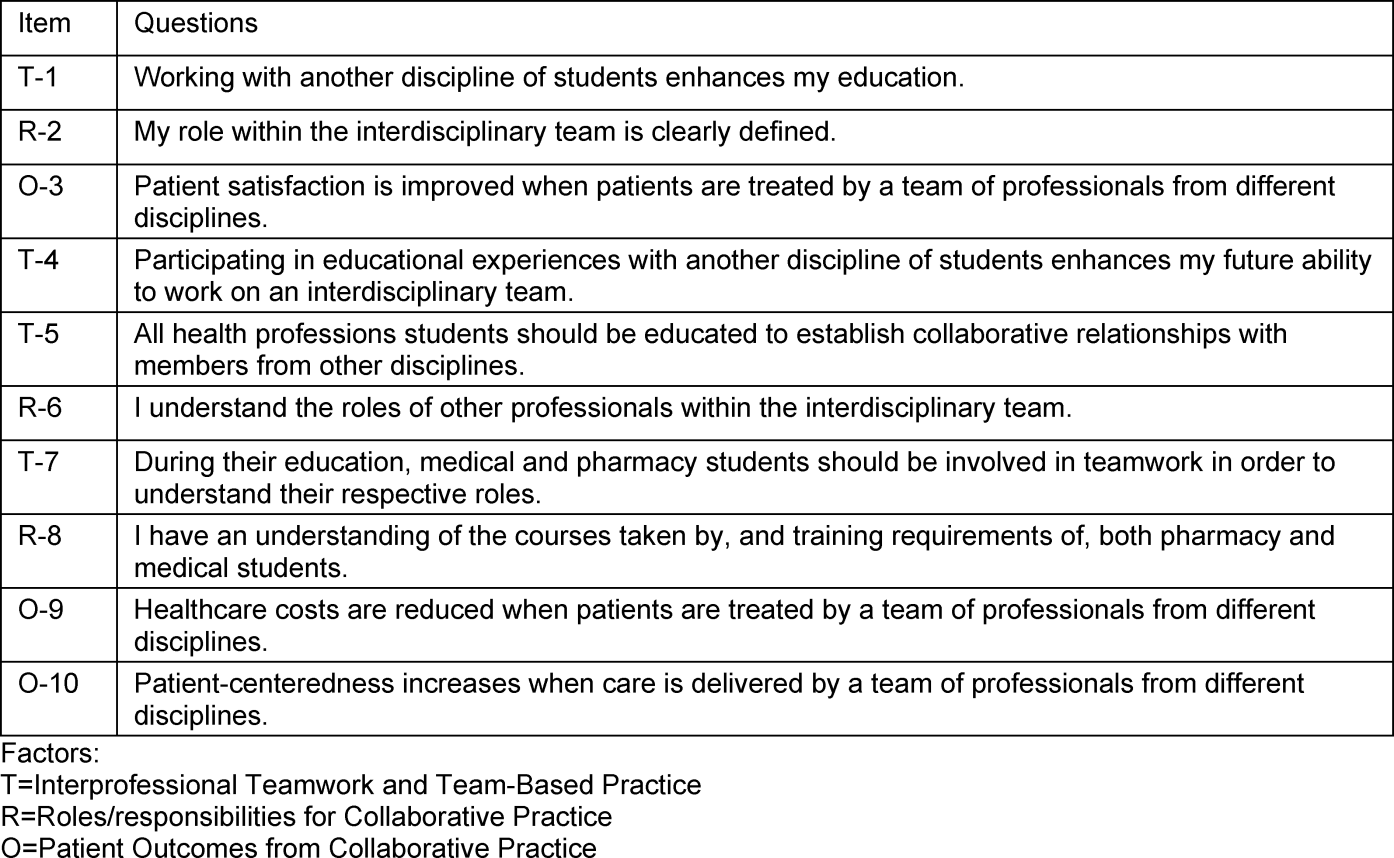
SPICE-2 questionnaire (Student Perceptions of Physician-Pharmacist Interprofessional Clinical Education, version 2)

**Table 3:**
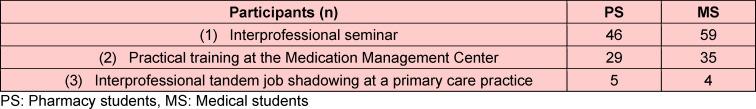
Participants in 3 cohorts of PILLE: 2020/21 winter semester, 2021 summer semester, 2021/22 winter semester (PS: pharmacy students, MS: medical students)
