# Supplementary material for: Development of a project for interprofessional collaboration between medical and pharmacy students to improve medication safety in polypharmacy (PILLE)
Source: GMS J Med Educ. 2023 Feb 15;40(1):Doc3. doi: 10.3205/zma001585 (PMC10010767; doi:10.3205/zma001585)
Supplement: Evaluation survey [file JME-40-3-s-001.pdf]

Attachment 1: Evaluation survey

Attachment 1 to Gehrke-Beck S, Peterson M, Herrmann WJ, Zimmermann N, Daub E, Seeger J, Schulz J, Czimmeck C, Lauterbach N, Peters H, Kloft C, Schulz M, Siebenbrodt I, Behrend R. *Development of a project for interprofessional collaboration between medical and pharmacy students to improve medication safety in polypharmacy (PILLE).* GMS J Med Educ. 2023;40(1):Doc3. DOI: 10.3025/zma001585

Evaluation of the interprofessional teaching and learning project "Polypharmacy - an interprofessional teaching and learning project" (PILLE) for pharmacy and medical students (Eva-PharMed) - Pre-Survey

Declaration of Consent

Voluntary participation:

You have the right to decline to participate in this survey without citing any reasons and without facing any negative repercussions. Participating in the survey at this time does not obligate you to continue your participation later. The respondents have the right to subsequently revoke their consent to participate voluntarily at any time.

General information on data protection:

Rights of the affected persons as stipulated under § 7 BlnDSG *(Berlin Data Protection Act)*

The legal basis for this study (survey) is your voluntary declaration of consent. By giving your consent, you agree to have your anonymously collected data processed for the purpose of conducting research on medical/pharmaceutical education. The study results could be published in the academic literature, whereby your identity will remain anonymous.

Your data will be collected and saved anonymously independently of the email address. No matching of the data with the email addresses will take place at any time. The collected data will only be connected to an ID number if you provide the necessary information to generate one. After the survey has been filled out, the dataset will be automatically saved. No individualized analysis will be performed. Your separately saved email address will only be used for communicating with you at the survey time points and to communicate the survey results. Third parties will not be given access to the data. After a period of 10 years the survey data will be destroyed in compliance with data protection law.

The survey respondents have the right to demand the erasure of the data at any time. This right may be exercised by contacting the person responsible for the project.

* I have read the information about the study, data protection and the voluntary participation.

- Yes
- No

* I declare that I am prepared to voluntarily participate in the study named above.

- Yes
- No

Evaluation of the interprofessional teaching and learning project "Polypharmacy - an interprofessional teaching and learning project" (PILLE) for pharmacy and medical students (Eva-PharMed) - Pre-Survey

Personal ID and sociodemographic information

First digit: Write down the *first letter* of *your mother's first name*.
Second digit: Write down the *second letter* of *your own first name.*
Third and fourth digits: Write down the *month* of *your own birthdate*. Use two digits!
(e.g., 09 for the birth month of September)
Fifth and sixth digits: Write down the *last two digits* of *your student enrollment number.*

Please write the resulting 6-digit personal ID comprised of letters and numbers:

Which subject are you studying?

- ◯ Medicine
- ◯ Pharmacy

Have you already completed professional training or another degree in healthcare?

- ◯ Yes
- ◯ No

What is your gender?

- ◯ Female
- ◯ Male
- ◯ Other
- ◯ No information given

Evaluation of the interprofessional teaching and learning project "Polypharmacy - an interprofessional teaching and learning project" (PILLE) for pharmacy and medical students (Eva-PharMed) - Pre-Survey

* Please evaluate the following statements:

	Strongly agree	Agree	Neutral	Disagree	Strongly disagree
Working with another discipline of students enhances my education.	○	○	○	○	○
My role within the interdisciplinary team is clearly defined.	○	○	○	○	○
Patient satisfaction is improved when patients are treated by a team of professionals from different disciplines.	○	○	○	○	○
Participating in educational experiences with another discipline of students enhances my future ability to work on an interdisciplinary team.	○	○	○	○	○
All health professions students should be educated to establish collaborative relationships with members from other disciplines.	○	○	○	○	○
I understand the roles of other professionals within the interdisciplinary team.	○	○	○	○	○
During their education, medical and pharmacy students should be involved in teamwork in order to understand their respective roles.	○	○	○	○	○

	Strongly agree	Agree	Neutral	Disagree	Strongly disagree
I have an understanding of the courses taken by, and training requirements of, both pharmacy and medical students.	○	○	○	○	○
Healthcare costs are reduced when patients are treated by a team of professionals from different disciplines.	○	○	○	○	○
Patient-centeredness increases when care is delivered by a team of professionals from different disciplines.	○	○	○	○	○

Evaluation of the interprofessional teaching and learning project "Polypharmacy - an interprofessional teaching and learning project" (PILLE) for pharmacy and medical students (Eva-PharMed) - Pre-Survey

What are your expectations of this interprofessional seminar?

What would you like yourself to have regarding interprofessional collaboration between the pharmacy and the primary care practice?

Evaluation of the interprofessional teaching and learning project "Polypharmacy - an interprofessional teaching and learning project" (PILLE) for pharmacy and medical students (Eva-PharMed) - Post Survey

Declaration of Consent

Voluntary participation:

You have the right to decline to participate in this survey without citing any reasons and without facing any negative repercussions. Participating in the survey at this time does not obligate you to continue your participation later.The respondents have the right to subsequently revoke their consent to participate voluntarily at any time.

General information on data protection:

Rights of the affected persons as stipulated under § 7 BlnDSG*(Berlin Data Protection Act)*

The legal basis for this study (survey) is your voluntary declaration of consent. By giving your consent, you agree to have your anonymously collected data processed for the purpose of conducting research on medical/pharmaceutical education. The study results could be published in the academic literature, whereby your identity will remain anonymous.

Your data will be collected and saved anonymously independently of the email address. No matching of the data with the email addresses will take place at any time. The collected data will only be connected to an ID number if you provide the necessary information to generate one. After the survey has been filled out, the dataset will be automatically saved. No individualized analysis will be performed. Your separately saved email address will only be used for communicating with you at the survey time points and to communicate the survey results. Third parties will not be given access to the data. After a period of 10 years the survey data will be destroyed in compliance with data protection law.

The survey respondents have the right to demand the erasure of the data at any time. This right may be exercised by contacting the person responsible for the project.

* I have read the information about the study, data protection and the voluntary participation.

- ◯ Yes
- ◯ No

* I declare that I am prepared to voluntarily participate in the study named above.

- ◯ Yes
- ◯ No

Evaluation of the interprofessional teaching and learning project "Polypharmacy - an interprofessional teaching and learning project" (PILLE) for pharmacy and medical students (Eva-PharMed) - Post Survey

Personal ID and sociodemographic information

First digit: Write down the *first letter* of *your mother's first name*.
Second digit: Write down the *second letter* of *your own first name.*
Third and fourth digits: Write down the *month* of *your own birthdate*. Use two digits!
(e.g., 09 for the birth month of September)
Fifth and sixth digits: Write down the *last two digits* of *your student enrollment number.*

Please write the resulting 6-digit personal ID comprised of letters and numbers:

* Which subject are you studying?

- Medicine
- Pharmacy

* Have you already completed professional training or another degree in healthcare?

- Yes
- No

* What is your gender?

- Female
- Male
- Other
- No information given

Evaluation of the interprofessional teaching and learning project "Polypharmacy - an interprofessional teaching and learning project" (PILLE) for pharmacy and medical students (Eva-PharMed) - Post Survey

* Please evaluate the following statements:

	Strongly agree	Agree		NeutralDisagr ee	Strongly disagree
Working with another discipline of students enhances my education.					
My role within the interdisciplinary team is clearly defined.					
Patient satisfaction is improved when patients are treated by a team of professionals from different disciplines.					
Participating in educational experiences with another discipline of students enhances my future ability to work on an interdisciplinary team.					
All health professions students should be educated to establish collaborative relationships with members from other disciplines.					
I understand the roles of other professionals within the interdisciplinary team.					
During their education, medical and pharmacy students should be involved in teamwork in order to understand their respective roles.					

	Strongly agree	Agree	Neutral	Disagree	Strongly disagree
I have an understanding of the courses taken by, and training requirements of, both pharmacy and medical students.	◯	◯	◯	◯	◯
Healthcare costs are reduced when patients are treated by a team of professionals from different disciplines.	◯	◯	◯	◯	◯
Patient-centeredness increases when care is delivered by a team of professionals from different disciplines.	◯	◯	◯	◯	◯

Evaluation of the interprofessional teaching and learning project "Polypharmacy - an interprofessional teaching and learning project" (PILLE) for pharmacy and medical students (Eva-PharMed) - Post Survey

What was for you the most important thing you learned about the other study program/profession?

How did your view of the other study program/profession change?

What do you want to put into practice in the collaboration between physicians and pharmacists in your future professional work?

Evaluation of the interprofessional teaching and learning project "Polypharmacy - an interprofessional teaching and learning project" (PILLE) for pharmacy and medical students (Eva-PharMed) - Post Survey

* Did you prepare yourself in advance for the interprofessional seminar?

- Yes, intensively
- Yes, briefly
- No

* Please evaluate the following statements:

	Strongly agree	Agree	Neutral	Disagree	Strongly disagree
I would have liked to have had more input / presentations.	○	○	○	○	○
I would have liked to have had more time for sharing / more time for small groups.	○	○	○	○	○

What I liked about the seminar:

What I wished had been different:

Evaluation of the interprofessional teaching and learning project "Polypharmacy - an interprofessional teaching and learning project" (PILLE) for pharmacy and medical students (Eva-PharMed) - Post Survey

* Please evaluation the following statement:

	Strongly agree	Agree	Neutral	Disagree	Strongly disagree
I would recommend the interprofessional seminar to others.	○	○	○	○	○
